# Supplementary material for: New Species and New Records of Otidea from China Based on Molecular and Morphological Data
Source: J Fungi (Basel). 2022 Mar 8;8(3):272. doi: 10.3390/jof8030272 (PMC8953430; doi:10.3390/jof8030272)
Supplement: Supplementary file 1 [file jof-08-00272-s001.zip › Supplementary Materials/Table S1_Xu al_Otidea from China.pdf]

Table S1. Information on sequences used in molecular phylogenetic analyses for *Otidea*.

Note: “—” shows no sequence in the GenBank database. GenBank accession numbers for sequences generated in this study are in boldface.

Species name	**Specimen voucher**	**Locality**	**ITS**	**LSU**	***ef1-α***	***rpb2***
Monascella botryosa	CBS 233.85	Spain	MH861870	MH873558	KC109256	JX943831
O. adorniae	MCVE 30102	Italy	MK850486	MK850502	—	—
O. adorniae	MCVE 30103	Italy	MK850485	MK850501	—	—
O. adorniae	MCVE 30106	Italy	MK850484	MK850500	—	—
O. adorniae	MCVE 30105	Italy	MK850483	MK850499	—	—
O. adorniae	MCVE 30104	Italy	MK850482	MK850498	—	—
O. alutacea	420526MF0389	China	**OM700202**	MG712347	—	—
O. alutacea	420526MF0402	China	—	MH141322	—	—
O. alutacea	S-F257085	Italy	KM010069	KM823192	KM823260	KM823386
O. alutacea	FLAS-F59409	Pakistan	MN495935	MN493146	—	—
O. alutacea	LAH35220	Pakistan	MN495934	MN493147	—	—
O. alutacea	GC 98092002	France	KM010073	KM823462	—	—
O. alutacea	KH.09.133	Norway	KM010071	KM823185	KM823253	KM823381
O. alutacea	ARAN A3023204	Spain	KM010072	KM823186	KM823254	KM823382
O. alutacea	HKAS 82112	China	KY498600	KY498605	—	—
O. alutacea	KS-94-111 (C)	Denmark	KM010074	KM823463	—	—
O. alutacea	S-F257084	Italy	KM010075	KM823464	—	—
O. alutacea	FLAS-F-62365	USA	MT373947	MT350446	—	—
O. alutacea	**HMAS 83560**	**China**	**OM701796**	DQ443442	**—**	**—**
O. alutacea	**HMJAU 22992**	**China**	**OM703442**	**OM722132**	**—**	**—**
O. alutacea	**HMAS 83559**	**China**	**OM704914**	DQ443441	—	—
O. alutacea	OSC 56777	USA	AF072071	AF086582	—	—
O. alutacea	OSC 56798	USA	AF072072	AF086583	—	—
O. alutacea	OSC 56747	USA	AF072070	KM823189	KM823257	—
O. alutacea	OSC 56770	USA	AF072073	AF086585	—	—
O. alutacea	KH.09.135	Norway	KM010064	KM823190	KM823258	KM823384
O. alutacea	KH.10.198	Sweden	KM010065	KM823459	—	—
O. alutacea	KH.13.50	Sweden	KM010076	KM823465	—	—
O. alutacea	JS.08.43	Sweden	KM010063	KM823458	—	—
O. alutacea	KH.09.178	Sweden	KM010066	KM823191	KM823259	KM823385
O. alutacea	K(M)142010	England	KT818924	—	—	—
O. alutacea	KS-94-192	Denmark	KM010067	KM823460	—	—
O. alutacea	Moorefun19	USA	KM010070	KM823194	KM823262	KM823387
O. alutacea	OSC 56782	USA	AF072076	AF086586	—	—
O. alutacea	OSC 56813	USA	AF072075	AF086584	—	—
O. alutacea	OSC 56758	USA	AF072074	KM823193	KM823261	—
O. alutacea	KH.07.46	Denmark	KM010061	KM823457	—	—
O. alutacea	KH.09.170	Sweden	KM010059	KC012691	KC109261	JX943830
O. alutacea	KH.10.193	Sweden	KM010060	KM823188	KM823256	—
O. alutacea	C-F-48045	Sweden	KM010068	KM823461	—	—
O. alutacea	WZ 2123 (HMAS 72058)	China	KU987013	KU987025	—	—
O. alutacea	WZ 2128 (HMAS 72057)	China	KU987014	KU987026	OM792147	OM792167
O. angusta	H6010804	Finland	KF717574	KM823195	KM823263	KM823388
O. apophysata	S-F257062	Germany	KM010077	KM823196	KM823264	KM823389

O. apophysata	K(M)87117	England	EU784382	—	—	—
O. aspera	**HMJAU 4166**	**China**	**OM743958**	**OM743964**	**OM792154**	**OM792174**
O. aspera	**HSA 278**	**China**	**OM743960**	**OM743951**	**OM792153**	**OM792175**
O. bicolor	HMAS 188415	China	KU987008	KU987020	—	—
O. bicolor	**HKAS 54453**	**China**	**OM714812**	**OM722133**	**OM792152**	**OM792173**
O. borealis	S-F242694	Finland	KM010023	KM823197	KM823265	KM823390
O. brevispora	HKAS 43003	China	—	DQ443450	—	—
O. brevispora	**HMAS 83551**	**China**	**OM792189**	**OM722134**	**OM792148**	**OM792168**
O. brunneoparva	KH.08.107	Sweden	KM010026	KM823200	KM823268	KM823393
O. brunneoparva	JS.08.66	Sweden	KM010028	KM823467	—	—
O. brunneoparva	KH.09.82	Sweden	KM010029	KM823198	KM823266	KM823391
O. brunneoparva	S-F249386	Finland	KM010024	KM823466	—	—
O. brunneoparva	S-F257086	Finland	KM010025	KM823199	KM823267	KM823392
O. brunneoparva	TUR-A 198582	Finland	KM010027	KM823468	—	—
O. bufonia	ZMU124	China	MW724241	—	—	—
O. bufonia	JS150904-08	Korea	KX963782	—	—	—
O. bufonia	JS.08.55	Sweden	KM010078	KM823469	—	—
O. bufonia	TUR-A 208341	Italy	MK850496	MK850509	—	—
O. bufonia	MCVE 29370	Italy	MG383802	MG383810	—	—
O. bufonia	TUR-A 148640	Italy	MK850492	MK850505	—	—
O. bufonia	MCVE 29367	Italy	MG383799	MG383807	—	—
O. bufonia	KH.07.37	Denmark	JN942767	JN941098	KC109262	JN993552
O. bufonia	KH.09.172	Sweden	JN942764	JN941097	KM823272	KM823397
O. bufonia	MCVE 29369	Italy	MG383801	MG383809	—	—
O. bufonia	MCVE 29368	Italy	MG383800	MG383808	—	—
O. bufonia	TUR-A 208338	Italy	MK850493	MK850506	—	—
O. bufonia	TUR-A 208342	Italy	MK850497	MK850510	—	—
O. bufonia	TUR-A 208339	Italy	MK850494	MK850507	—	—
O. bufonia	KH.09.248	Spain	JN942766	JN941084	KM823269	KM823394
O. bufonia	KH.09.249	France	KM010079	KM823201	KM823271	KM823396
O. bufonia	NV 2009.11.01	France	JN942765	JN941085	KM823270	KM823395
O. bufonia	**HMAS 188416**	**China**	**OM716927**	**OM722136**	**—**	**—**
O. bufonia	**HKAS 54451**	**China**	**OM716929**	**OM722137**	**—**	**—**
O. bufonia	**HMAS 76106**	**China**	**OM716928**	**OM723194**	**—**	**—**
O. bufonia	MCVE 29372	Finland	MG383803	MG383812	—	—
O. bufonia	TUR-A 208340	Italy	MK850495	MK850508	—	—
O. aff. *bufonia*	K(M)41595	England	MN627811	—	—	—
O. aff. *bufonia*	K(M)156077	England	MN627813	—	—	—
O. cf. *bufonia*	SE-2015	USA	KT275680	—	—	—
O. cf. *bufonia*	GO-2009-365	Mexico	KC152161	—	—	—
O. caeruleopruinosa	H6010805	Finland	KF717575	KM823202	KM823273	KM823398
O. caeruleopruinosa	KH.13.48	Sweden	KM010081	KM823470	—	—
O. caeruleopruinosa	MT 10082601	Spain	KM010030	KM823203	—	KM823399
O. cantharella	JS.08.47	Sweden	KM010083	KM823472	—	—
O. cantharella	JS.08.18	Sweden	KM010082	KM823471	—	—
O. cantharella	KH.09.125	Sweden	KM010084	KM823205	KM823274	KM823401
O. cantharella	NV 2008.09.16	France	KM010085	KM823204	—	KM823400
O. concinna	JS.08.59	Sweden	KM010031	KM823473	—	—
O. concinna	KH.09.250	Spain	JN942775	JN941095	KM823276	KM823403

O. concinna	KH.09.183	Sweden	KM010032	JN941089	KM823275	KM823402
O. cupulata	**HSA 406**	**China**	**OM743959**	**OM743970**	**OM792165**	**OM792177**
O. cupulata	**HSA 218**	**China**	**OM743973**	**OM743954**	**OM792166**	**OM792176**
O. daliensis	SEST-06081702	Spain	KM010086	KM823206	KM823277	KM823404
O. daliensis	AM-AR17-016	Argentina	MH930311	—	—	—
O. daliensis	HMAS 57688	China	—	DQ443445	—	—
O. felina	9572	Italy	JF908509	—	—	—
O. filiformis	**HMAS 188468**	**China**	**OM743949**	**OM743955**	**OM792155**	**OM792178**
O. filiformis	**BJTC C505**	**China**	**MW554241**	**OM743968**	**OM792156**	**OM792179**
O. filiformis	**BJTC L482**	**China**	**MW554466**	**OM743963**	**OM792157**	**OM792180**
O. flavidobrunneola	H6010806	Finland	KF717576	KM823209	KM823279	KM823407
O. flavidobrunneola	KH.09.153	Norway	KM010088	KM823207	—	KM823405
O. flavidobrunneola	H6010830	Finland	KM010087	KM823208	KM823278	KM823406
O. formicarum	KH.11.104	Sweden	KM010033	KM823475	—	—
O. formicarum	JS.08.63	Sweden	KM010035	KM823212	KM823282	—
O. formicarum	H6003549	Finland	KF717577	KM823211	KM823281	KM823409
O. formicarum	H6003550	Finland	KM010036	KM823474	—	—
O. formicarum	S-F244372	Norway	KM010034	KM823210	KM823280	KM823408
O. hanseniae	WZ 2202	China	KU987012	KU987024	KU987033	—
O. hanseniae	XF007	China	KU987016	KU987028	KU987035	KU987038
O. integra	S-F108342	Italy	KP006504	—	—	—
O. kauffmanii	MICH 14409	USA	KF717579	—	—	—
O. kaushalii	T. Læssøe 6236	Malaysia	KM010119	AF335111	KM823326	KM823455
O. khakicolorata	**BJTC FM107**	**China**	**OM743967**	**OM743950**	**OM792158**	**OM792181**
O. korfii	Z.W. Ge 1913	China	KU987017	KU987029	KU987036	—
O. kunmingensis	HKAS 49452	China	MK850489	—	—	—
O. lactea	HMAS 61359	China	**OM721661**	DQ443447	—	—
O. leporina	JS.08.46	Sweden	KM010089	KM823477	—	—
O. leporina	JS.08.92	Sweden	KM010091	KM823478	—	—
O. leporina	NV 2008.09.28	France	KM010092	KM823214	KM823284	KM823411
O. leporina	OSC 56824	USA	—	KM823216	KM823286	KM823413
O. leporina	OSC 56784	USA	—	KM823215	KM823285	KM823412
O. leporina	H6003548	Finland	KF717578	KM823222	KM823292	KM823421
O. leporina	PA12	Latvia	KR019792	—	—	—
O. leporina	KH.09.93	Sweden	KM010090	KM823213	KM823283	KM823410
O. leporina	**HMAS 254161**	**China**	**OM731674**	**OM727116**	**—**	**—**
O. leporina	**HMAS 83570**	**China**	**OM721662**	DQ443443	—	—
O. leporina	**HMAS 29549**	**China**	**OM721663**	**OM727117**	**—**	**—**
O. microspora	AH30502	USA	AF072094	—	—	—
O. minor	KH.98.84	Denmark	KM010041	KM823217	KM823287	KM823414
O. minor	H6008618	Finland	KM010039	KM823219	KM823289	KM823416
O. minor	KH.10.311	Sweden	KM010042	KM823218	KM823288	KM823415
O. minor	H6003841	Finland	KM010040	KM823479	—	—
O. minor	TL-Vorsø-0754	Denmark	KM010043	KM823480	—	—
O. minor	C-F-83445	Denmark	KM010038	KM823481	—	—
O. minor	CL 950914-01	Italy	KM010044	KM823220	—	—
O. mirabilis	KH.09.188	Sweden	JN942770	JN941086	—	KM823417
O. mirabilis	MCVE 29374	Italy	MG383805	MG383813	—	—
O. mirabilis	GMFN 1951	Italy	KF717580	KM823483	—	—

O. mirabilis	KH.01.09	Denmark	JN942769	AY500540	KM823290	KM823419
O. mirabilis	S-F257083	Finland	KM010095	KM823482	—	—
O. mirabilis	NV 2008.09.14	France	JN942768	JN941094	KM823291	KM823420
O. mirabilis	KH.10.285	Sweden	KM010094	KM823221	—	KM823418
O. mirabilis	**HMAS 83568**	**China**	**OM721670**	DQ443449	**—**	**—**
O. mirabilis	**HKAS 28129**	**China**	**OM721671**	**OM728199**	**—**	**—**
O. mirabilis	**HKAS 33633**	**China**	**OM721672**	**OM728197**	**—**	**—**
O. mirabilis	**HKAS 30708**	**China**	**OM721673**	**OM728195**	**—**	**—**
O. mirabilis	**BJTC FM292**	**China**	**OM721674**	**OM728196**	**—**	**—**
O. mirabilis	**HKAS 37272**	**China**	**OM721676**	**OM728200**	**—**	**—**
O. mirabilis	**HSA 234**	**China**	**OM721675**	**OM728198**	**—**	**—**
O. mirabilis	**HMJAU 26926**	**China**	**OM721677**	**OM728283**	**—**	**—**
O. nannfeldtii	**BJTC FM168**	**China**	**OM721808**	**OM728519**	**—**	**—**
O. nannfeldtii	**BJTC FM169**	**China**	**OM721807**	**OM728521**	**—**	**—**
O. nannfeldtii	**BJTC FM170**	**China**	**OM721810**	**OM728520**	**—**	**—**
O. nannfeldtii	**BJTC FM236**	**China**	**OM721811**	**OM728522**	**—**	**—**
O. nannfeldtii	**BJTC FM243**	**China**	**OM721809**	**OM728523**	**—**	**—**
O. nannfeldtii	CL091116-17	Italy	KM010096	KM823484	—	—
O. nannfeldtii	S-F257096	Italy	KM010097	KM823485	—	—
O. nannfeldtii	CL091207-01	Italy	KM010098	KM823486	—	—
O. nannfeldtii	JS.08.103	Sweden	KM010045	KM823224	KM823294	KM823423
O. nannfeldtii	S-F249387	Finland	KM010093	KM823225	—	—
O. nannfeldtii	H6002902	Finland	KF717581	KM823228	KM823297	KM823426
O. nannfeldtii	NV 2008.10.01	France	KM010099	KM823227	KM823296	KM823425
O. nannfeldtii	rh101310	USA	KM010100	KM823226	KM823295	KM823424
O. nannfeldtii	KH.10.302	Sweden	KM010101	KM823223	KM823293	KM823422
O. nannfeldtii	**HMAS 83573**	**China**	**OM721812**	**OM728524**	**—**	**—**
O. olivaceobrunnea	HMAS 23948	China	KU987010	KU987022	—	—
O. onotica	OSC 56734	USA	AF072066	AF086577	—	—
O. onotica	OSC 56759	USA	AF072068	JN941088	KM823300	KM823430
O. onotica	OSC 56801	USA	AF072067	AF086578	—	—
O. onotica	C-F-89691	Denmark	JN942773	JN941090	—	KM823427
O. onotica	JS.08.48	Sweden	KM010102	KM823487	—	—
O. onotica	MCVE 23277	Italy	KM010104	KM823488	—	—
O. onotica	KH.10.284	Sweden	KP006505	KM823229	KM823299	KM823429
O. onotica	KH.09.132	Norway	KM010103	KC012692	KC109263	JX943828
O. onotica	KH.09.136	Norway	JN942772	JN941096	KM823298	KM823428
O. onotica	DMS-9327924	Denmark	MT644895	MT644895	—	—
O. onotica	**HMAS 280314**	**China**	**OM721813**	**OM728528**	**—**	**—**
O. onotica	**HMAS 33473**	**China**	**OM721814**	**OM728529**	**—**	**—**
O. onotica	**HMAS 97555**	**China**	**OM721817**	**OM728532**	—	—
O. oregonensis	OSC 56745	USA	AF072089	KM823232	KM823303	KM823433
O. oregonensis	NSW 6354	USA	AF072088	AF086598	—	—
O. oregonensis	OSC 56829	USA	AF072087	AF086597	—	—
O. oregonensis	Moorefun 58	USA	KM010048	KM823231	KM823302	KM823432
O. oregonensis	rh139	USA	KM010046	KM823489	—	—
O. oregonensis	Moorefun 31	USA	KM010047	KM823230	KM823301	KM823431
O. papillata	TUR 102134	Finland	KM010105	KM823233	KM823304	KM823434
O. papillata	H6003547	Finland	KF717582	KM823234	KM823305	KM823435

O. papillata	741	USA	KX389120	—	—	—
O. papillata f. *pallidefurfuracea*	NV 2007.09.27	France	KF717584	KM823235	—	—
O. parvispora	K(M)70199	England	EU784380	—	—	—
O. parvispora	JS.08.81	Sweden	KM010062	KM823187	KM823255	KM823383
O. parvispora	MCVE 30108	Spain	MK850491	MK850504	—	—
O. parvispora	MCVE 30107	Greece	MK850490	MK850503	—	—
O. parvula	**BJTC FM210-A**	**China**	**OM743965**	**OM743966**	**OM792159**	**OM792182**
O. parvula	**BJTC FM210-B**	**China**	**OM743962**	**OM743956**	**OM792160**	**OM792183**
O. phlebophora	JV06-385	Denmark	KM010049	KM823236	KM823306	KM823436
O. phlebophora	S-F108338	Sweden	KM010050	KM823490	—	—
O. phlebophora	K(M)143475	England	MN627790	—	—	—
O. platyspora	KH.09.163	Sweden	KM010106	KM823238	KM823308	KM823438
O. platyspora	HK0846	Sweden	KM010107	KM823491	—	—
O. platyspora	JV06-656	Denmark	KM010108	KM823237	KM823307	KM823437
O. plicara	**BJTC FM262-A**	**China**	**OM743957**	**OM743971**	**OM792161**	**OM792187**
O. plicara	**BJTC FM262-B**	**China**	**OM743961**	**OM743952**	**OM792162**	**OM792186**
O. propinquata	**BJTC FM171**	**China**	**OM731678**	**OM728537**	—	—
O. propinquata	**BJTC FM49**	**China**	**OM731677**	**OM728536**	—	—
O. propinquata	**BJTC FM233**	**China**	**OM731673**	**OM728545**	—	—
O. propinquata	JS.08.67	Sweden	KM010110	KM823492	—	—
O. propinquata	NV 2008.09.15	France	KM010111	KM823240	KM823310	KM823440
O. propinquata	HMAS 83564	China	KU987009	KU987021	—	—
O. propinquata	KH.09.99	Sweden	KM010109	KM823239	KM823309	KM823439
O. propinquata	**HMAS 83575**	**China**	**OM731675**	**OM728547**	**—**	**—**
O. propinquata	**HMAS 83576**	**China**	**OM731676**	**OM728546**	**—**	**—**
O. pruinosa	MFLU:16-0611	China	KY498604	KY498609	—	—
O. pruinosa	HKAS 81819	China	KY498602	KY498607	**OM792149**	**OM792169**
O. pseudoformicarum	HKAS 101386	China	KY498601	KY498606	—	MG980709
O. pseudoleporina	Moorefun14	USA	KM010113	KM823242	KM823312	KM823442
O. pseudoleporina	rh101910	USA	KM010112	KM823243	KM823313	KM823443
O. pseudoleporina	NSW7574	USA	AF072083	AF086593	—	—
O. pseudoleporina	OSC 56749	USA	AF072082	AF086592	—	—
O. pseudoleporina	OSC 56809	USA	AF072080	KM823241	KM823311	KM823441
O. pseudoleporina	OSC 56760	USA	AF072081	KM823244	KM823314	KM823444
O. purpureobrunnea	**BJTC FM1061**	**China**	**OM743969**	**OM743972**	**OM792163**	**OM792185**
O. purpureobrunnea	**BJTC FM1048**	**China**	**OM743974**	**OM743953**	**OM792164**	**OM792184**
O. purpurea	HKAS 5670	China	—	MG742413	—	—
O. purpureogrisea	Z.W. Ge 863 (HKAS 449358)	China	KU987011	KU987023	KU987032	KU987037
O. purpureogrisea	WZ 2157 (HMAS72805)	China	KU987015	KU987027	KU987034	**OM792170**
O. rainierensis	A.H. Smith 30553 (MICH)	USA	KF717583	KM823245	KM823315	KM823445
O. saliceticola	MCVE 29365	Italy	MG383806	MG383814	—	—
O. sinensis	HMAS 61360	China	**OM722037**	DQ443451	—	—
O. smithii	OSC 56811	USA	AF072060	AF086572	—	—
O. smithii	OSC 56799	USA	AF072063	JN941087	KM823317	KM823447
O. smithii	OSC 56753	USA	AF072062	AF086574	—	—
O. smithii	OSC 56823	USA	EU669213	EU669265	—	—
O. smithii	ecv3345	USA	JN942771	JN941093	KM823316	KM823446

O. stipitata	HKAS 87865	China	KY498603	KY498608	—	—
O. subformicarum	CMP 1179	Spain	KM010053	KM823246	KM823318	KM823448
O. subformicarum	CL050928-30	Italy	KM010052	KM823247	KM823319	KM823449
O. subformicarum	S-F256979	Spain	KM010051	KM823494	—	—
O. subformicarum	S-F242696	Spain	KM010054	KM823495	—	—
O. aff. *subformicarum*	FH301035	Mexico	KM010055	KM823249	KM823321	KM823451
O. aff. *subformicarum*	FH301036	Mexico	KM010056	KM823248	KM823320	KM823450
O. subpurpurea	HKAS 54449	China	KU987018	KU987030	**OM792150**	**OM792171**
O. subpurpurea	HMAS 97530	China	KU987019	KU987031	—	—
O. subpurpurea	**HKAS 54944**	**China**	**OM722058**	**OM728605**	**OM792151**	**OM792172**
O. subterranea	RH69	USA	FJ404767	FJ404767	—	—
O. subterranea	RH97	USA	FJ404766	FJ404766	—	—
O. tuomikoskii	JS.08.100	Sweden	KM010116	KM823498	—	—
O. tuomikoskii	NV 2008.09.08	France	JN942777	JN941091	KM823323	KM823453
O. tuomikoskii	KH.11.77	Sweden	KM010117	KM823499	—	—
O. tuomikoskii	KH.09.130	Norway	JN942776	JN941092	KM823322	KM823452
O. tuomikoskii	OSC 56826	USA	AF072086	AF086596	—	—
O. tuomikoskii	OSC 56756	USA	AF072084	AF086594	—	—
O. tuomikoskii	OSC 56761	USA	AF072085	KM823251	KM823325	KM823454
O. tuomikoskii	H6002901	Finland	KF717585	KM823250	KM823324	—
O. tuomikoskii	MK200065	Sweden	KM010115	KM823497	—	—
O. tuomikoskii	JS.08.68	Sweden	KM010114	KM823496	—	—
O. tuomikoskii	PA13	Latvia	KR019793	—	—	—
O. unicisa	FH RH1666	USA	MT373948	MT373948	—	—
O. unicisa	ZW Geo65-Clark	USA	KM010118	AY789369	—	—
O. unicisa	TENN 066839	USA	MG677140	—	—	—
O. unicisa	KH.06.06	USA	—	KC012693	KC109264	JX943829
O. yunnanensis	HMAS 82166	China	—	DQ443452	—	—
O. sp.	LM4111	Germany	KM576487	—	—	—
O. sp.	216-318	USA	MH038148	—	—	—
O. sp.	M540	China	MW551141	MW520022	—	—
O. sp.	LM16	Spain	KM576486	—	—	—
O. sp.	GMFN 2293	Italy	KM010037	KM823476	—	—
O. sp.	LM3580	Germany	KM576485	—	—	—
O. sp.	B250	Estonia	FN669231	—	—	—
O. sp.	Plot3_33_M1	Slovenia	MW028106	—	—	—
O. sp.	MK1081	Sweden	KM010058	KM823501	—	—
O. sp.	MK0942	Sweden	KM010057	KM823500	—	—
O. sp.	KH.09.79	Sweden	KM010120	KM823252	KM823327	KM823456
O. sp.	TENN 070359	USA	KY777379	—	—	—
O. sp.	FLAS-F-64209	USA	MT373946	MT350445	—	—
O. sp.	RH1218	USA	MN653026	—	—	—
O. sp.	FLAS-F-60735	USA	MH571410	—	—	—
Uncultured fungus	RM D12	Switzerland	KX886079	—	—	—
Uncultured fungus	F104	Poland	MH834496	—	—	—
Uncultured fungus	I1	Poland	MG835426	—	—	—
Warcupia terrestris	CBS 891.69	Canada	MH859473	MH871254	KC109308	JX943832
